# Dose-Response Association of Uncontrolled Blood Pressure and Cardiovascular Disease Risk Factors with Hyperuricemia and Gout

**DOI:** 10.1371/journal.pone.0056546

**Published:** 2013-02-27

**Authors:** Stephen P. Juraschek, Lara C. Kovell, Edgar R. Miller, Allan C. Gelber

**Affiliations:** 1 Department of Epidemiology, Johns Hopkins Bloomberg School of Public Health, Baltimore, Maryland, United States of America; 2 Welch Center for Prevention, Epidemiology and Clinical Research, Baltimore, Maryland, United States of America; 3 Department of Medicine, Johns Hopkins University School of Medicine, Baltimore, Maryland, United States of America; Virginia Commonwealth University, United States of America

## Abstract

**Background:**

First-line therapy of hypertension includes diuretics, known to exert a multiplicative increase on the risk of gout. Detailed insight into the underlying prevalence of hyperuricemia and gout in persons with uncontrolled blood pressure (BP) and common comorbidities is informative to practitioners initiating antihypertensive agents. We quantify the prevalence of hyperuricemia and gout in persons with uncontrolled BP and additional cardiovascular disease (CVD) risk factors.

**Methods and Findings:**

We performed a cross-sectional study of non-institutionalized US adults, 18 years and older, using the National Health and Nutrition Examination Surveys in 1988–1994 and 1999–2010. Hyperuricemia was defined as serum uric acid >6.0 mg/dL in women; >7.0 mg/dL in men. Gout was ascertained by self-report of physician-diagnosed gout. Uncontrolled BP was based on measured systolic BP≥140 mmHg and diastolic BP≥90 mmHg. Additional CVD risk factors included obesity, reduced glomerular filtration rate, and dyslipidemia. The prevalence of hyperuricemia was 6–8% among healthy US adults, 10–15% among adults with uncontrolled BP, 22–25% with uncontrolled BP and one additional CVD risk factor, and 34–37% with uncontrolled BP and two additional CVD risk factors. Similarly, the prevalence of gout was successively greater, at 1–2%, 4–5%, 6–8%, and 8–12%, respectively, across these same health status categories. In 2007–2010, those with uncontrolled BP and 2 additional CVD risk factors compared to those without CVD risk factors had prevalence ratios of 4.5 (95% CI 3.5–5.6) and 4.5 (95% CI: 3.1–6.3) for hyperuricemia and gout respectively (*P*<0.01).

**Conclusions:**

Health care providers should be cognizant of the incrementally higher prevalence of hyperuricemia and gout among patients with uncontrolled BP and additional CVD risk factors. With one in three people affected by hyperuricemia among those with several CVD risk factors, physicians should consider their anti-hypertensive regimens carefully and potentially screen for hyperuricemia or gout.

## Introduction

Hyperuricemia is a well-known mediator of gout [1], an acute, incapacitating form of arthritis that incurs great human suffering and health-related expense [2], [3]. Hypertension, the most commonly diagnosed condition during ambulatory visits in the United States [4], is a recognized risk factor of both hyperuricemia [5] and gout [6]–[8]. First-line therapy for drug treatment of hypertension includes thiazide diuretics, known to exert a multiplicative increase on the risk of gout [9]. While hypertensive status guides initiation of blood pressure (BP) lowering therapies, detailed insight into the underlying prevalence of hyperuricemia and gout in persons presenting with normal, prehypertensive, and progressive hypertensive stages has yet to be described.

Patients rarely present to their primary physicians with hypertension as their sole health condition. In fact, approximately 70% of all hypertensive patients have at least one other chronic condition [4]. The presence of the metabolic syndrome, which includes elevated BP, has frequently been cited as a risk factor for both hyperuricemia and gout [10]–[12]. However, patients may present with one or two components of the syndrome, failing to meet its full criteria. Furthermore, some traditional cardiovascular disease (CVD) risk factors such as reduced glomerular filtration rate (GFR) [13] are not included in the metabolic syndrome definition. Together these issues limit the utility of a syndrome approach to stratifying risk for hyperuricemia and gout in the clinical setting. Rather, determination of those individual CVD risk factors associated with prevalent hyperuricemia and gout would further inform healthcare providers of underlying risk.

The purpose of the present study was to examine the hypothesis that uncontrolled BP together with additional CVD risk factors are associated in a dose-response fashion with prevalent hyperuricemia and gout. Furthermore, we evaluate the incremental association between various degrees of BP and other CVD risk factors with prevalent hyperuricemia and gout. These objectives were achieved using the National Health and Nutrition Examination Survey (NHANES) in 1988–1994, 1999–2002, 2003–2006, and 2007–2010.

## Materials and Methods

### Study Population

The NHANES surveys are large, cross-sectional studies conducted by the National Center for Health Statistics (NCHS), using a complex multistage sampling design. Specifically, the surveys examined in the present report include NHANES III, conducted in 1988–1994, and four-year intervals of the continuous NHANES: conducted in the years 1999–2002, 2003–2006, and 2007–2010. Our analysis was restricted to the interviews, physical examinations, and laboratory measurements gathered by the NHANES mobile examination centers from participants, age 18 years and older, with a measurement of serum uric acid. These studies were approved by the NCHS Research Ethics Review Board; informed consent was obtained from all participants [14], [15].

### Uncontrolled Blood Pressure and Additional Cardiovascular Disease Risk Factors

Uncontrolled BP was defined as a systolic blood pressure (SBP) ≥140 mmHg or diastolic blood pressure (DBP) ≥90 mmHg [16] regardless of hypertension status or preexisting antihypertensive medication use. BP was determined by averaging 1–4 mercury sphygmomanometer measurements, depending on the maximum number available [14], [15]. Categories of BP were based on the Seventh Report of the Joint National Committee on Prevention, Detection, Evaluation, and Treatment of High Blood Pressure (JNC VII), as follows: normal (SBP<120 mmHg and DBP<80 mmHg); prehypertensive (SBP between 120–139 mmHg or DBP between 80–89 mmHg), hypertension stage I (SBP between 140–159 mmHg or DBP between 90–99 mmHg), and hypertension stage II (SBP≥160 mmHg or DBP≥100 mmHg) [17].

Additional CVD risk factors evaluated for potential inclusion in our models were: elevated body mass index (BMI), low GFR, reduced high density lipoprotein (HDL) cholesterol, high total cholesterol, high glycated hemoglobin, and smoking status. BMI based on weight and standing height measurements was categorized using the World Health Organization classification system as follows: underweight (<18.5 kg/m^2^), normal (18.5–24.9 kg/m^2^), overweight (25–29.9 kg/m^2^), obesity class I (30–34.9 kg/m^2^) and obesity classes II or III (35 kg/m^2^ and greater) [18], [19]. GFR was estimated with the Chronic Kidney Disease Epidemiology Collaboration equation [20], using standardized serum creatinine measurements [21], and was classified as follows: ≥90 mL/min per 1.73 m^2^, 60–89 mL/min per 1.73 m^2^, 30–59 mL/min per 1.73 m^2^, and 15–29 mL/min per 1.73 m^2^ [22]. While not excluded from our study population, survey participants with an estimated GFR <15 mL/min per 1.73 m^2^ are not presented as a distinct stratum due to small sample size.

HDL cholesterol and total cholesterol categories were based on the National Health Lung and Blood Institute Adult Treatment Panel III [23]. HDL cholesterol was categorized as high (≥60 mg/dL for both genders), intermediate (between 40–59 mg/dL for men and 50–59 mg/dL for women), and low (<40 mg/dL for men and <50 mg/dL for women). Total cholesterol was categorized as desirable (<200 mg/dL), borderline high (200–239 mg/dL) and high (≥240 mg/dL). Category of diabetes was determined using glycated hemoglobin [24], [25]. Hemoglobin A1c was subsequently classified as normal (<5.7%), prediabetic (5.7–6.4%), and diabetic (≥6.5%), following guidelines established by the American Diabetes Association [26]. Self-reported smoking was divided into 3 categories: never, former, and current.

### Hyperuricemia and Gout

Hyperuricemia was defined as a serum uric acid measurement >6.0 mg/dL (360 µmol/L) in women or >7.0 mg/dL (420 µmol/L) in men [11]. Uric acid was measured via an oxidation reaction, involving uricase and peroxidase [14], [15]. Gout was considered present if participants responded “yes” to the question, “Has a doctor or other health professional ever told you that you had gout?” (NHANES 2007–10) or “Has a doctor ever told you that you had gout?” (NHANES III). In a sensitivity analysis, we utilized a more specific definition of gout, requiring either hyperuricemia or self-reported use of gout medication, in addition to self-reported gout.

### Other Covariates

Age, gender, and race/ethnicity were uniformly recorded. Race/ethnicity was categorized as non-Hispanic white, non-Hispanic black, Mexican American, and other, following the classification established in NHANES III. Relevant medication use was dichotomized (yes or no) for gout medications (allopurinol, probenecid, colchicine, sulfinpyrazone, and alloxanthine), thiazide diuretics (hydrochlorothiazide, chlorothiazide, chlorthalidone, indapamide, metolazone, bendroflumethiazide, methyclothiazide, and hydroflumethiazide), and any diuretic including thiazides (loop diuretics, potassium-sparing diuretics, thiazide diuretics, carbonic anhydrase inhibitors, or miscellaneous diuretics). Finally, alcohol consumption was categorized as never, former, current non-excessive, or current excessive, using NHANES-derived definitions [27].

### Statistical Analyses

All analyses were performed in concordance with the NHANES complex sampling design using the sample weights, primary sampling units, and strata accompanying each survey. NHANES III (1988–1994) were weighted using the provided 6-year weights. For the continuous surveys, years 1999–2002 were weighted using the provided 4-year weights, while 2-year weights in years 2003–2006 and 2007–2010 were combined as recommended [14], [15]. Due to the absence of gout ascertainment during the continuous NHANES 1999–2006 survey periods, data on gout status were only available from NHANES 2007–2008 and 2009–2010. Standard errors for all estimates were calculated using the recommended Taylor series (linearization) method [14], [15]. Analyses were performed using Stata 11.1 (StataCorp LP, College Station, TX).

Weighted prevalence estimates, or means and their associated standard errors, were calculated for demographic characteristics, CVD risk factors, use of gout and diuretic medication, and alcohol use for each of the survey periods. In addition, we determined the prevalence and prevalence ratio (PR) of hyperuricemia according to the number of applicable CVD risk factors during each period. Risk factors with a consistent dose-response pattern were plotted to facilitate visual demonstration and comparison of trends, and were incorporated into models evaluating the concomitant presence of uncontrolled BP (i.e. hypertension stages I or II) with mean serum uric acid level, the prevalence of hyperuricemia, and the prevalence of gout. For each NHANES participant, the number of CVD risk factors was based on the presence of the following: eGFR <60 mL/min per 1.73 m^2^, BMI ≥30 kg/m^2^, HDL <40 mg/dL in men or <50 mg/dL in women, or total cholesterol ≥240 mg/dL. Additional CVD risk factor categories ranged from 0–2. Persons with 3–4 CVD risk factors were too few in number to conduct meaningful stratum-specific analyses.

The difference in serum uric acid between categories of risk factors was calculated using linear regression models with adjustment for age, gender, and race/ethnicity. PRs for hyperuricemia and gout were determined via Poisson regression models, adjusted for age, gender, and race/ethnicity. We also performed a sensitivity analysis in which uncontrolled BP was considered as one of the possible CVD risk factors, rather than as a prerequisite condition. Furthermore, we conducted stratified analyses by gender or race/ethnicity to determine whether the association between uncontrolled BP and additional CVD risk factors was modified by demographic characteristics. Finally, we performed a sensitivity analysis with adjustment for diuretic use to determine if pharmacologic treatment contributed toward associations between uncontrolled BP and gout.

### Results

There were 16,171 adults, age 18 and older, examined at the mobile examination center in NHANES 1988–1994, 9,836 in NHANES 1999–2002, 9,943 in NHANES 2003–2006, and 11,526 in NHANES 2007–2010 among whom serum uric acid was measured (**Table 1**). The prevalence of hyperuricemia was greatest in the last survey period (18.3% or 38.7 million adults). Similarly, the prevalence of gout was 2.62% in 1988–1994 compared with 3.75% in 2007–2010, corresponding to about 4.6 million and 7.7 million adults, respectively.

**Table 1:** Population Characteristics of US Adults Aged 18 Years and Older According to NHANES Survey Period, 1988–1994 &1990–2010.

	1988–1994, No. = 247,729,796*		1999–2002, No. = 278,652,243*		2003–2006, No. = 288,919,825*		2007–2010, No. = 299,539,907*	
	No.†	Weighted Mean or % (SE)	No.†	Weighted Mean or % (SE)	No.†	Weighted Mean or % (SE)	No.†	Weighted Mean or % (SE)
Age, Mean	16,171	43.87 (0.45)	9,836	45.07 (0.34)	9,943	45.59 (0.47)	11,526	46.09 (0.32)
Gender, %								
Male	8,579	47.85 (0.44)	5,147	48.21 (0.40)	5,134	48.35 (0.43)	5,866	48.50 (0.43)
Female	7,592	52.15 (0.44)	4,689	51.79 (0.40)	4,809	51.65 (0.43)	5,660	51.50 (0.43)
Race/Ethnicity, %								
Non-Hispanic white	6,629	76.49 (1.28)	4,636	71.76 (1.81)	4,927	72.49 (2.20)	5,485	69.14 (2.48)
Non-Hispanic black	4,412	10.54 (0.60)	1,897	10.38 (1.18)	2,229	11.23 (1.33)	2,103	10.60 (1.00)
Mexican American	4,480	5.18 (0.42)	2,528	7.22 (0.89)	2,121	7.98 (1.10)	2,120	8.66 (1.34)
Other‡	650	7.80 (0.83)	775	10.64 (1.75)	666	8.29 (0.78)	1,818	11.60 (1.25)
Blood Pressure (mmHg), %								
Normal (SBP<120 or DBP<80)	6,747	47.42 (0.94)	4,173	45.10 (1.01)	4,416	46.59 (0.87)	5,123	49.72 (0.94)
Prehypertensive (SBP: 120–139 or DBP: 80–89)	5,618	34.80 (0.58)	3,236	36.40 (0.63)	3,216	36.24 (0.64)	3,928	35.72 (0.80)
Stage 1 HTN (SBP: 140–159 or DBP: 90–99)	2,600	13.14 (0.52)	1,353	12.87 (0.65)	1,219	12.45 (0.60)	1,470	11.24 (0.37)
Stage 2 HTN (SBP≥160 or DBP≥100)	1,169	4.65 (0.26)	730	5.63 (0.33)	589	4.72 (0.27)	542	3.31 (0.16)
Body Mass Index (kg/m^2^), %								
Below weight (<18.5)	357	2.50 (0.22)	188	2.12 (0.19)	180	1.80 (0.16)	199	1.80 (0.19)
Normal (18.5–24.9)	6,170	43.04 (0.89)	3,146	33.88 (0.80)	3,073	32.07 (0.84)	3,190	30.46 (0.75)
Overweight (25–29.9)	5,569	32.49 (0.56)	3,322	34.39 (0.82)	3,299	33.36 (0.68)	3,855	33.72 (0.62)
Obese Class I (30–34.9)	2,596	14.34(0.42)	1,707	17.52 (0.58)	1,902	19.27 (0.51)	2,347	19.49 (0.46)
Obese Classes II & III (≥35)	1,442	7.63 (0.51)	1,168	12.09 (0.67)	1,323	13.49 (0.63)	1,783	14.52 (0.46)
Estimated GFR (mL/min per 1.73 m^2^), %								
≥90	10,952	70.28 (0.93)	6,096	60.30 (0.95)	5,874	56.66 (1.37)	7,040	62.88 (1.10)
60–89	4,099	24.99 (0.79)	2,917	33.27 (0.91)	3,090	35.92 (1.06)	3,485	30.75 (0.90)
30–59	1,033	4.43 (0.26)	734	5.87 (0.30)	881	6.81 (0.45)	887	5.75 (0.30)
15–29	58	0.24 (0.04)	53	0.33 (0.07)	79	0.49 (0.08)	81	0.48 (0.06)
HDL Cholesterol (mg/dL), %								
High (Men or Women ≥60)	3,958	24.30 (0.89)	2,499	24.90 (0.88)	3,258	31.99 (0.70)	3,232	28.73 (0.79)
Intermediate (Men 40–59; Women 50–59)	6,223	39.16 (0.90)	3,863	38.86 (0.56)	4,124	41.31 (0.66)	4,459	38.42 (0.48)
Low (Men <40, Women <50)	5,838	36.54 (1.05)	3,455	36.24 (0.99)	2,557	26.70 (0.72)	3,834	32.85 (0.78)
Total Cholesterol (mg/dL), %								
Desirable (<200)	7,942	50.15 (0.93)	5,117	51.28 (0.87)	5,553	53.87 (0.83)	6,679	57.17 (0.73)
Borderline High (200–239)	4,987	30.74 (0.76)	3,069	31.95 (0.60)	2,862	30.17 (0.74)	3,277	29.13 (0.64)
High (≥240)	3,200	19.11 (0.61)	1,632	16.77 (0.63)	1,525	15.96 (0.48)	1,569	13.70 (0.53)
Hemoglobin A1c (%), %								
Normal (<5.7)	12,282	84.33 (0.82)	8,010	85.58 (0.73)	7,959	84.04 (0.58)	7,889	75.37 (0.82)
Prediabetes (5.7–6.4)	2,487	10.51 (0.63)	1,037	8.57 (0.55)	1,169	9.81 (0.40)	2,384	17.17 (0.57)
Diabetes (≥6.5)	1,329	5.16 (0.30)	783	5.85 (0.35)	795	6.15 (0.32)	1,225	7.45 (0.40)
Smoking Status, %								
Never	8,164	46.55 (0.76)	4,570	50.43 (1.29)	4,573	50.19 (0.81)	5,829	54.05 (1.15)
Former	3,894	25.26 (0.61)	2,341	25.34 (0.96)	2,352	25.19 (0.66)	2,745	24.55 (0.71)
Current	4,113	28.19 (0.84)	1,892	24.23 (0.87)	1,984	24.62 (0.84)	2,415	21.40 (0.79)
Gout, %	444	2.62 (0.19)	NA	NA	NA	NA	514	3.75 (0.25)
Serum Uric Acid (mg/dL), Mean	16,171	5.32 (0.02)	9,836	5.36 (0.02)	9,943	5.37 (0.02)	11,526	5.46 (0.02)
Hyperuricemia, %§	2,781	15.56 (0.48)	1,732	17.68 (0.73)	1,634	16.64 (0.60)	2,206	18.32 (0.57)
Diuretics, %	1,342	6.42 (0.33)	836	7.64 (0.52)	1,122	9.89 (0.59)	1,392	9.92 (0.47)
Thiazides, %	405	1.87 (0.15)	354	3.24 (0.30)	594	5.43 (0.35)	771	5.53 (0.32)
Gout Medication Use, %∥	148	0.92 (0.11)	98	0.98 (0.12)	107	0.90 (0.11)	179	1.30 (0.17)
Alcohol status, %								
Never	2,941	13.69 (0.76)	1,268	12.97 (1.67)	1,169	11.44 (0.82)	1,379	11.07 (0.57)
Former	5,614	32.01 (1.01)	2,314	24.38 (0.84)	2,394	24.82 (1.03)	2,660	22.81 (1.04)
Non-excessive current	3,116	24.07 (0.95)	2,720	34.64 (1.47)	2,772	35.78 (1.08)	3,351	36.05 (0.85)
Excessive current	3,894	30.24 (1.18)	1,975	28.01 (0.73)	1,918	27.96 (1.08)	2,622	30.07 (0.87)

Abbreviations: BP, blood pressure; SBP, systolic blood pressure; DBP, diastolic blood pressure; HTN, hypertension; HDL, high density lipoprotein; GFR, glomerular filtration rate; NA, not available.

* Weighted number.

† The unweighted number (for means) or numerator (for prevalences) corresponding with each variable category.

‡ In order to account for a change in NHANES race/ethnicity definitions in 2005–2010, we placed Hispanic in the “Other” to be consistent with NHANES 1988–2004.

§ Defined as >6.0 mg/dL (360 µmol/L) in women and >7.0 mg/dL (420 µmol/L) in men.

∥ Gout medications included allopurinol, probenecid, colchicine, sulfinpyrazone, alloxanthine.

**Figure 1:**
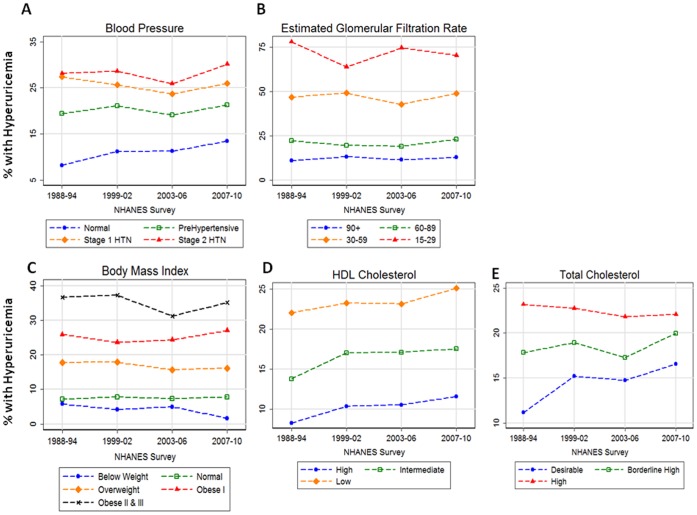
The prevalence of hyperuricemia (%) stratified by degree of cardiovascular disease risk factor, namely, blood pressure, estimated glomerular filtration rate (mL/min per 1.73 m^2^), body mass index, high-density lipoprotein (HDL) cholesterol, and total cholesterol. Specific category details may be found in the Methods. Y-axes units vary by risk factor.

Measured BP, BMI, eGFR, HDL cholesterol, and total cholesterol each demonstrated a graded association with the prevalence of hyperuricemia in all survey periods (**Figure 1**), even after adjustment for the other risk factors (**Supplemental Table S1, Table S2, Table S3, TableS4**). The prevalence of hyperuricemia was about 8–11% among individuals with a normal BP compared with 26–30% in individuals with hypertension stage II, corresponding to adjusted PRs ranging from 1.41 to 1.60 (*P*≤0.005). Similarly, the prevalence of hyperuricemia among participants who were obese class II or III was 31–37% compared to 7–8% among participants with a normal BMI, with adjusted PRs ranging from 3.5 to 3.9 (*P*<0.001). Estimated GFR showed an even greater gradation in hyperuricemia prevalence with lower renal function, being 11–13% among individuals with an eGFR ≥90 mL/min per 1.73 m^2^ versus 64–78% among individuals with an eGFR between 15 and 29 mL/min per 1.73 m^2^. Similarly, the prevalence of hyperuricemia was as high as 20–25% in the lowest HDL or highest total cholesterol categories.

In contrast, we did not observe a successively greater prevalence of hyperuricemia with higher levels of hemoglobin A1c or smoking status (**Supplemental Table S1, Table S2, Table S3, TableS4**). Rather, both elevated hemoglobin A1c (≥6.5%) and current smoking status were associated with less prominent PRs compared with their respective intermediate categories (prediabetes or former smoking, respectively); these two CVD risk factors were not included in subsequent models because they lacked a clear dose-response relationship.

In all survey periods, normotensive participants lacking other CVD risk factors had the lowest mean serum uric acid concentrations of about 4.9–5.0 mg/dL (290–300 µmol/L) (**Table 2**). Notably, mean serum uric acid values were greater among participants with uncontrolled BP, though they were only significant in NHANES 1988–1994 and 1999–2002. However, the presence of one additional CVD risk factor with uncontrolled BP was associated with a higher mean serum uric acid concentration, ranging from 0.6–0.8 mg/dL (*P*<0.01) in each of the four NHANES survey periods. Likewise, among participants with uncontrolled BP and two additional CVD risk factors the mean serum uric acid concentration ranged between 6.1 and 6.3 mg/dL, representing a difference of at least 1.2 mg/dL (*P*<0.01). These elevations in serum uric acid were consistent with a greater prevalence of hyperuricemia across these same categories.

**Table 2:** Mean Serum Uric Acid and Prevalence of Hyperuricemia according to Uncontrolled Blood Pressure and Number of Cardiovascular Disease Risk Factors, 1988–1994 & 1999–2010.

	Unweighted No.*		Mean SUA (SE)		SUA β†		*P*		Prevalence (SE)	Prevalence Ratio†	*P*
NHANES 1988–1994											
Healthy‡	5,487		4.92 (0.03)		Ref		Ref		6.60 (0.49)	Ref	Ref
Uncontrolled BP Alone§	973		5.40 (0.08)		0.31 (0.15, 0.48)		<0.01		15.33 (1.92)	1.85 (1.35, 2.54)	<0.01
Plus 1 CVD Risk Factor∥	1,488		5.75 (0.06)		0.76 (0.62, 0.89)		<0.01		22.83 (1.62)	2.86 (2.29, 3.57)	<0.01
Plus 2 CVD Risk Factors	986		6.26 (0.06)		1.37 (1.22, 1.52)		<0.01		36.77 (2.30)	4.87 (3.91, 6.07)	<0.01
NHANES 1999–2002											
Healthy‡	3,111		4.96 (0.03)		Ref		Ref		8.10 (0.54)	Ref	Ref
Uncontrolled BP Alone§	584		5.19 (0.07)		0.19 (0.06, 0.32)		0.01		15.55 (2.04)	1.76 (1.30, 2.37)	<0.01
Plus 1 CVD Risk Factor∥	782		5.71 (0.08)		0.79 (0.63, 0.95)		<0.01		24.62 (2.37)	2.86 (2.22, 3.68)	<0.01
Plus 2 CVD Risk Factors	574		6.10 (0.09)		1.25 (1.04, 1.45)		<0.01		35.07 (2.81)	4.23 (3.30, 5.42)	<0.01
NHANES 2003–2006											
Healthy‡		3,399		4.98 (0.02)		Ref		Ref	7.08 (0.66)	Ref	Ref
Uncontrolled BP Alone§		526		5.09 (0.07)		0.08 (−0.06, 0.23)		0.25	10.15 (1.61)	1.28 (0.93, 1.75)	0.12
Plus 1 CVD Risk Factor∥		761		5.74 (0.07)		0.69 (0.53, 0.85)		<0.01	24.90 (2.27)	3.08 (2.28, 4.16)	<0.01
Plus 2 CVD Risk Factors		416		6.15¶		1.20 (1.00, 1.39)		<0.01	34.94¶	4.52 (3.33, 6.14)	<0.01
NHANES 2007–2010											
Healthy‡	3,645		5.02 (0.03)		Ref		Ref		7.67 (0.63)	Ref	Ref
Uncontrolled BP Alone§	570		5.35 (0.10)		0.08 (−0.09, 0.25)		0.33		15.32 (2.11)	1.60 (1.09, 2.35)	0.02
Plus1 CVD Risk Factor∥	785		5.72 (0.06)		0.61 (0.48, 0.74)		<0.01		24.48 (2.14)	2.69 (2.11, 3.44)	<0.01
Plus 2 CVD Risk Factors	528		6.31¶		1.29 (1.09, 1.49)		<0.01		37.84¶	4.45 (3.52, 5.63)	<0.01

Abbreviations: SUA, serum uric acid; BP, blood pressure; CVD, cardiovascular disease.

* The unweighted total number of people (denominator) available in each category.

† Adjusted for age, gender, and race/ethnicity.

‡ Healthy is defined as the absence of uncontrolled blood pressure and any of the 4 cardiovascular disease risk factors associated with serum uric acid.

§ Uncontrolled blood pressure, defined as a systolic blood pressure ≥140 mmHg or diastolic blood pressure ≥90 mmHg, with no additional cardiovascular disease risk factors.

∥ A cardiovascular disease risk factor is defined as any of the following: estimated glomerular filtration rate <60 mL/min per 1.73 m2, body mass index ≥30 kg/m2, high density lipoprotein <40 mg/dL (1.04 mmol/L) in men or <50 mg/dL (1.30 mmol/L) in women, or total cholesterol ≥240 mg/dL (6.22 mmol/L).

¶ Unable to estimate variance due to small sample size in multiple weighting strata.

Gout prevalence was lowest among individuals who were free of uncontrolled BP or any additional CVD risk factor, ranging from 1–2% (**Table 3**). In contrast, the prevalence of gout was ∼4–5% among individuals with uncontrolled BP, although the PRs were not statistically significant in all survey periods. With the presence of one additional CVD risk factor, above and beyond uncontrolled BP, the prevalence of gout was 6–8%, representing a 1.9–3.2 fold greater association. Moreover, when gout was examined in individuals with uncontrolled BP and two other CVD risk factors, the prevalence of gout rose higher still, to 7–12%, and was 3.4–5.9 times greater than the prevalence in the healthy group (all *P*-values <0.01). A sensitivity analysis restricting gout to individuals with hyperuricemia or self-reported gout medication use yielded virtually the same findings (**Supplemental Table S5**).

**Table 3:** Prevalence of Gout according to Uncontrolled Blood Pressure and Number of Cardiovascular Disease Risk Factors, 1988–1994 & 2007–2010.

	Unweighted No.*	Prevalence (SE)	Prevalence Ratio†	*P*
NHANES 1988–1994				
Healthy‡	5,486	1.03 (0.25)	Ref	Ref
Uncontrolled BP Alone§	973	4.44 (0.80)	1.82 (1.08, 3.09)	0.03
Plus 1 CVD Risk Factor∥	1,488	5.96 (0.99)	2.56 (1.51, 4.34)	<0.01
Plus 2 CVD Risk Factors	986	7.72 (1.36)	3.80 (2.20, 6.57)	<0.01
NHANES 2007–2008				
Healthy‡	1,603	1.29 (0.21)	Ref	Ref
Uncontrolled BP Alone§	291	5.45 (1.57)	1.89 (0.75, 4.77)	0.16
Plus 1 CVD Risk Factor∥	405	7.72 (1.49)	3.20 (1.49, 6.90)	0.01
Plus 2 CVD Risk Factors	264	12.00 (1.79)	5.86 (3.20, 10.73)	<0.01
NHANES 2009–2010				
Healthy‡	1,721	1.68 (0.22)	Ref	Ref
Uncontrolled BP Alone§	273	4.13 (1.49)	1.12 (0.43, 2.91)	0.80
Plus 1 CVD Risk Factor∥	378	6.96 (1.49)	1.86 (0.99, 3.47)	0.05
Plus 2 CVD Risk Factors	259	9.77¶	3.38 (2.32, 4.93)	<0.01
NHANES 2007–2010				
Healthy‡	3,324	1.49 (0.15)	Ref	Ref
Uncontrolled BP Alone§	564	4.81 (1.07)	1.47 (0.79, 2.72)	0.21
Plus 1 CVD Risk Factor∥	783	7.36 (1.06)	2.41 (1.52, 3.85)	<0.01
Plus 2 CVD Risk Factors	523	10.92¶	4.45 (3.13, 6.32)	<0.01

Abbreviations: BP, blood pressure; CVD, cardiovascular disease.

* The unweighted total number of people (denominator) available in each category.

† Adjusted for age, gender, and race/ethnicity.

‡ Healthy is defined as the absence of uncontrolled blood pressure and any of the 4 cardiovascular disease risk factors associated with serum uric acid.

§ Uncontrolled blood pressure, defined as a systolic blood pressure ≥140 mmHg or diastolic blood pressure ≥90 mmHg, with no additional cardiovascular disease risk factors.

∥ A cardiovascular disease risk factor is defined as any of the following: estimated glomerular filtration rate <60 mL/min per 1.73 m2, body mass index ≥30 kg/m2, high density lipoprotein <40 mg/dL (1.04 mmol/L) in men or <50 mg/dL (1.30 mmol/L) in women, or total cholesterol ≥240 mg/dL (6.22 mmol/L).

¶ Unable to estimate variance due to small sample size in multiple weighting strata.

A sensitivity analysis including uncontrolled BP as one of the CVD risk factors did not fundamentally change our findings (**Supplemental Table S6, Table S7**). Similarly, we did not find evidence of effect modification by strata of gender or race/ethnicity (**Supplemental Table S8**). Furthermore, adjustment for diuretic use did not alter the observed associations between uncontrolled BP and additional CVD risk factors (results not shown).

## Discussion

We found a strong, incremental elevation in the prevalence of hyperuricemia and gout with increasing levels of BP, BMI, and total cholesterol, and with decreasing levels of estimated GFR and HDL cholesterol. Notably, regardless of survey period, individuals with uncontrolled BP and two additional CVD risk factors demonstrated a 4-fold or greater prevalence of hyperuricemia, and at least a 3-fold or greater prevalence of gout compared to normotensive individuals without these risk factors.

Our results are consistent with prior studies demonstrating an association between hyperuricemia or gout and the metabolic syndrome [10]–[12]. However, metabolic syndrome, in its aggregate formulation, may not be the most suitable exposure for evaluating gout prevalence. Whereas many CVD risk factors are components of the metabolic syndrome and associate positively with hyperuricemia and gout, one component - serum glucose concentration - exhibits an inverse relationship at high concentrations [5], [28]–[32]. We observed this inverse association with greater levels of hemoglobin A1c. Furthermore, other CVD risk factors, such as reduced eGFR, are not among the characteristics comprising metabolic syndrome [33]. As a result, our findings suggest that gout prevalence may be optimally characterized in relation to individual and additional CVD risk factors rather than as an aggregated syndrome. Moreover, these component factors are both intuitive and readily ascertained in the ambulatory care setting.

Hypertension is strongly associated with serum uric acid [11]. In our analysis, the prevalence of hyperuricemia among individuals with uncontrolled BP, in the absence of other CVD risk factors, was ∼10–15% compared to 7–8% in healthy individuals. Furthermore, in each survey period, we observed an incrementally greater prevalence of hyperuricemia with each successive stage of BP. The underlying mechanism is unknown. Some report that hyperuricemia is not an independent risk factor for hypertension [34], while other laboratory [35] and epidemiologic studies suggest that uric acid plays a causal role in the development of hypertension [36], [37]. Furthermore, prospective studies describe hypertension as a risk factor for hyperuricemia [5] and of gout [6]–[8]. Regardless of mechanism, it is clear that hypertension and hyperuricemia are positively related, with about a quarter of all individuals with a blood pressure in the hypertensive range (i.e. uncontrolled blood pressure) also meeting criteria for hyperuricemia (**Supplemental Table S1, Table S2, Table S3, Table S4**).

Hypertension is frequently present with other health conditions [4]. CVD risk factors, including kidney disease, dyslipidemia, and obesity, along with hypertension, are among the most common reasons for ambulatory medical visits in the US [38]. Our analyses indicate that each of these risk factors is individually and incrementally associated with a greater prevalence of hyperuricemia and gout (**Figure 1** **and Table S1, Table S2, Table S3, Table S4**). Furthermore, the addition of two risk factors was sufficient for the prevalence of hyperuricemia and gout to be as high as 35% and 7% of the American population, respectively.

In 2008, hypertension was diagnosed in about 46,000 ambulatory visits in the US, making it the most commonly diagnosed condition [38]. Based on our prevalence estimates, roughly 10,000 (15%) of these visits included individuals with hyperuricemia. Treating physicians are likely to initiate a thiazide agent as a first-line antihypertensive per the JNC VII guidelines [17]. In fact, our analysis suggests that thiazide use has increased from 1.5% to 5.6% over the past 20 years. New thiazide use has been shown to increase serum urate levels by 0.55 mg/dL and increase the risk of gout by 44% [9]. We recognize that widespread public health benefit can be achieved by small, marginal reductions in CVD risk factors [39]. Conversely, population-based increases in serum uric acid, mediated by the use of thiazide and other diuretic agents, may have a detrimental effect on the burden of gout in the US population. Given the high prevalence of hyperuricemia among hypertensive individuals, particularly in those with additional CVD risk factors, it is important that primary care providers be aware of these associations, especially when evaluating a patient with concomitant joint pain and swelling. Furthermore, future BP management guidelines should consider screening for hyperuricemia and gout in candidates with a thiazide indication, as well as discuss alternative medications with fewer serum urate-related effects [40].

This study has a number of important limitations. Although NHANES is well-suited for describing the prevalence of hyperuricemia and gout in the US population, its cross-sectional design does not account for temporality, limiting causal inference. Furthermore, in this study, gout was ascertained via survey question rather than a clinical diagnosis, which precludes synovial fluid analysis for detection of confirmatory urate crystals [41]. Although a crystal-proven diagnosis is the gold standard diagnostic approach in clinical practice, self-report is a reliable [42] and practical tool in the context of epidemiologic research. Nevertheless, when in a sensitivity analysis, we restricted the outcome of self-reported, physician-diagnosed gout to those NHANES participants with hyperuricemia or receiving urate-lowering agents, the findings were unchanged.

The prevalence of hyperuricemia and gout is substantially and significantly greater among individuals with uncontrolled BP and additional CVD risk factors. When initiating medical therapy and in subsequent follow-up visits for hypertensive patients, particularly those harboring one or more additional CVD risk factors, health care providers should be vigilant for underlying hyperuricemia and risk of gout.

## Supporting Information

Table S1 **Prevalence of Hyperuricemia by Level of Cardiovascular Disease Risk Factor in NHANES III (1988–1994).**
(DOCX)

Table S2 **Prevalence of Hyperuricemia by Level of Cardiovascular Disease Risk Factor in NHANES 1999–2002.**
(DOCX)

Table S3 **Prevalence of Hyperuricemia by Level of Cardiovascular Disease Risk Factor in NHANES 2003–2006.**
(DOCX)

Table S4 **Prevalence of Hyperuricemia by Level of Cardiovascular Disease Risk Factor in NHANES 2007–2010.**
(DOCX)

Table S5 **Prevalence of Gout Defined by Self-Report and Either Hyperuricemia or Gout Medication Use According to Number of Cardiovascular Disease Risk Factors.**
(DOCX)

Table S6 **Prevalence of Hyperuricemia According to Number of Cardiovascular Disease Risk Factors.**
(DOCX)

Table S7 **Prevalence of Gout According to Number of Cardiovascular Disease Risk Factors.**
(DOCX)

Table S8 **Prevalence Ratios of Gout by Strata of Gender or Race/Ethnicity in NHANES 1988–1994 & 2007–2010.**
(DOCX)
