# Supplementary material for: Dose-Response Association of Uncontrolled Blood Pressure and Cardiovascular Disease Risk Factors with Hyperuricemia and Gout
Source: PLoS One. 2013 Feb 27;8(2):e56546. doi: 10.1371/journal.pone.0056546 (PMC3584090; doi:10.1371/journal.pone.0056546)
Supplement: Table S1 — Prevalence of Hyperuricemia by Level of Cardiovascular Disease Risk Factor in NHANES III (1988–1994). (DOCX) [file pone.0056546.s001.docx]

| **Supplemental Table S1. Prevalence of Hyperuricemia by Level of Cardiovascular Disease Risk Factor in NHANES III (1988-1994)** | | | | | | | |
| --- | --- | --- | --- | --- | --- | --- | --- |
|  | |  |  | NHANES III (1988-1994) | |  | |
|  | |  | Unweighted No. | Prevalence, % (SE) | Partially Adjusted Prevalence Ratio (95% CI)* | Fully Adjusted Prevalence Ratio (95% CI)† | |
| Blood Pressure (mmHg) | | |  |  |  |  | |
|  | | SBP<120 or DBP<80 | 6,747 | 8.22 (0.52) | Ref | Ref | |
|  | | SBP: 120-139 or DBP: 80-89 | 5,618 | 19.45 (0.83) | 1.94 (1.62, 2.33) | 1.49 (1.26, 1.78) | |
|  | | SBP: 140-159 or DBP: 90-99 | 2,600 | 27.38 (0.93) | 2.44 (2.00, 2.98) | 1.66 (1.39, 1.97) | |
|  | | SBP ≥160 or DBP ≥100 | 1,169 | 28.17 (2.20) | 2.38 (1.87, 3.04) | 1.55 (1.23, 1.97) | |
| Body Mass Index (kg/m^2^) | | |  |  |  |  | |
|  | | <18.5 | 357 | 5.78 (2.06) | 0.91 (0.44, 1.89) | 1.04 (0.48, 2.23) | |
|  | | 18.5-24.9 | 6,170 | 7.18 (0.37) | Ref | Ref | |
|  | | 25-29.9 | 5,569 | 17.83 (0.63) | 2.09 (1.83, 2.38) | 1.77 (1.57, 2.01) | |
|  | | 30-34.9 | 2,596 | 25.94 (1.58) | 3.16 (2.69, 3.71) | 2.45 (2.11, 2.86) | |
|  | | ≥35 | 1,442 | 36.69 (1.86) | 5.03 (4.45, 5.68) | 3.60 (3.19, 4.07) | |
| Estimated GFR (mL/min per 1.73m^2^) | | |  |  |  |  | |
|  | | ≥90 | 10,952 | 10.98 (0.55) | Ref | Ref | |
|  | | 60-89 | 4,099 | 22.17 (0.78) | 1.93 (1.63, 2.27) | 1.76 (1.52, 2.04) | |
|  | | 30-59 | 1,033 | 46.88 (1.95) | 4.00 (3.27, 4.90) | 3.56 (2.95, 4.30) | |
|  | | 15-29 | 58 | 78.03‡ | 6.50 (4.76, 8.87) | 6.13 (4.57, 8.22) | |
| HDL Cholesterol (mg/dL) | | |  |  |  |  | |
|  | | Men or Women ≥60 | 3,958 | 8.28 (0.57) | Ref | Ref | |
|  | | Men 40-59; Women 50-59 | 6,223 | 13.80 (0.80) | 1.53 (1.28, 1.83) | 1.28 (1.09, 1.50) | |
|  | | Men <40, Women <50 | 5,838 | 22.05 (0.70) | 2.53 (2.22, 2.88) | 1.73 (1.54, 1.95) | |
| Total Cholesterol (mg/dL) | | |  |  |  |  | |
|  | | <200 | 7,942 | 11.17 (0.62) | Ref | Ref | |
|  | | 200-239 | 4,987 | 17.83 (0.75) | 1.37 (1.23, 1.53) | 1.21 (1.08, 1.36) | |
|  | | ≥240 | 3,200 | 23.19 (0.75) | 1.70 (1.53, 1.89) | 1.40 (1.24, 1.58) | |
| Hemoglobin A1c (%), % | | |  |  |  |  | |
|  | | Normal (<5.7) | 12,282 | 13.42 (0.46) | Ref | Ref | |
|  | | Prediabetes (5.7-6.4) | 2,487 | 28.29 (1.26) | 1.57 (1.37, 1.80) | 1.16 (1.03, 1.31) | |
|  | | Diabetes (≥6.5) | 1,329 | 24.38‡ | 1.30 (1.08, 1.56) | 0.77 (0.64, 0.94) | |
| Smoking Status, % | | |  |  |  |  | |
|  | | Never | 8,164 | 14.47 (0.74) | Ref | Ref | |
|  | | Former | 3,894 | 20.05 (1.08) | 1.08 (0.91, 1.28) | 1.00 (0.85, 1.19) | |
|  | | Current | 4,113 | 13.34 (0.71) | 0.93 (0.79, 1.08) | 0.98 (0.84, 1.15) | |
| Abbreviations: GFR, glomerular filtration rate; HDL, high density lipoprotein | | | | | | | |
| *Adjusted for age, gender, and race/ethnicity | | | |  |  |  | |
| †Adjusted for age, gender, race/ethnicity, blood pressure level, estimated GFR, body mass index level, HDL cholesterol level, total cholesterol level, hemoglobin A1c, and smoking status | | | | | | | |
| ‡Unable to estimate variance due to inadequate sample size | | | | | | | |
|  | | | | | |  |  |
