# Supplementary material for: Dose-Response Association of Uncontrolled Blood Pressure and Cardiovascular Disease Risk Factors with Hyperuricemia and Gout
Source: PLoS One. 2013 Feb 27;8(2):e56546. doi: 10.1371/journal.pone.0056546 (PMC3584090; doi:10.1371/journal.pone.0056546)
Supplement: Table S2 — Prevalence of Hyperuricemia by Level of Cardiovascular Disease Risk Factor in NHANES 1999–2002. (DOCX) [file pone.0056546.s002.docx]

|  | | | | | | |  |
| --- | --- | --- | --- | --- | --- | --- | --- |
| **Supplemental Table S2. Prevalence of Hyperuricemia by Level of Cardiovascular Disease Risk Factor in NHANES 1999-2002** | | | | | | | |
|  |  |  | NHANES 1999-2002 | |  | | |
|  |  | Unweighted No. | Prevalence, % (SE) | Partially Adjusted Prevalence Ratio (95% CI)* | Fully Adjusted Prevalence Ratio (95% CI)† | | |
| Blood Pressure (mmHg) | |  |  |  |  | | |
|  | SBP<120 or DBP<80 | 4,173 | 11.22 (0.79) | Ref | Ref | | |
|  | SBP: 120-139 or DBP: 80-89 | 3,236 | 21.12 (1.00) | 1.62 (1.38, 1.90) | 1.42 (1.20, 1.66) | | |
|  | SBP: 140-159 or DBP: 90-99 | 1,353 | 25.66 (1.60) | 1.85 (1.56, 2.19) | 1.48 (1.26, 1.74) | | |
|  | SBP≥160 or DBP ≥100 | 730 | 28.64 (2.20) | 2.04 (1.62, 2.58) | 1.59 (1.21, 2.10) | | |
| Body Mass Index (kg/m^2^) | |  |  |  |  | | |
|  | <18.5 | 188 | 4.17 (1.56) | 0.62 (0.29, 1.35) | 0.72 (0.34, 1.50) | | |
|  | 18.5-24.9 | 3,146 | 7.80 (0.73) | Ref | Ref | | |
|  | 25-29.9 | 3,322 | 17.86 (0.82) | 2.05 (1.72, 2.43) | 1.89 (1.60, 2.24) | | |
|  | 30-34.9 | 1,707 | 23.58 (1.39) | 2.81 (2.27, 3.48) | 2.33 (1.84, 2.94) | | |
|  | ≥35 | 1,168 | 37.26 (2.08) | 4.84 (3.98, 5.88) | 3.98 (3.17, 5.01) | | |
| Estimated GFR (mL/min per 1.73m^2^) | |  |  |  |  | | |
|  | ≥90 | 6,096 | 13.25 (0.81) | Ref | Ref | | |
|  | 60-89 | 2,917 | 19.59 (0.90) | 1.45 (1.22, 1.73) | 1.44 (1.22, 1.70) | | |
|  | 30-59 | 734 | 49.07 (2.21) | 3.80 (3.02, 4.80) | 3.78 (3.18, 4.48) | | |
|  | 15-29 | 53 | 63.93‡ | 4.59 (3.05, 6.89) | 3.47 (2.33, 5.17) | | |
| HDL Cholesterol (mg/dL) | |  |  |  |  | | |
|  | Men or Women ≥60 | 2,499 | 10.38 (0.82) | Ref | Ref | | |
|  | Men 40-59; Women 50-59 | 3,863 | 17.05 (0.84) | 1.49 (1.23, 1.79) | 1.14 (0.94, 1.39) | | |
|  | Men <40, Women <50 | 3,455 | 23.26 (1.27) | 2.23 (1.80, 2.76) | 1.46 (1.17, 1.83) | | |
| Total Cholesterol (mg/dL) | |  |  |  |  | | |
|  | <200 | 5,117 | 15.20 (0.75) | Ref | Ref | | |
|  | 200-239 | 3,069 | 18.93 (1.00) | 1.14 (1.03, 1.25) | 1.14 (1.01, 1.28) | | |
|  | ≥240 | 1,632 | 22.76 (1.39) | 1.35 (1.18, 1.55) | 1.27 (1.14, 1.42) | | |
| Hemoglobin A1c (%), % | |  |  |  |  | | |
|  | Normal (<5.7) | 8,010 | 15.89 (0.66) | Ref | Ref | | |
|  | Prediabetes (5.7-6.4) | 1,037 | 31.26 (2.55) | 1.61 (1.36, 1.91) | 1.19 (1.00, 1.41) | | |
|  | Diabetes (≥6.5) | 783 | 22.90 (2.25) | 1.16 (0.95, 1.42) | 0.82 (0.64, 1.04) | | |
| Smoking Status, % | |  |  |  |  | | |
|  | Never | 4,570 | 16.79 (0.96) | Ref | Ref | | |
|  | Former | 2,341 | 21.88 (1.09) | 1.08 (0.97, 1.20) | 1.08 (0.96, 1.20) | | |
|  | Current | 1,892 | 15.32 (1.07) | 0.91 (0.78, 1.07) | 1.05 (0.91, 1.22) | | |
| Abbreviations: GFR, glomerular filtration rate; HDL, high density lipoprotein | | | | |  | | |
| *Adjusted for age, gender, and race/ethnicity | | | | | |  |  |
| †Adjusted for age, gender, race/ethnicity, blood pressure level, estimated GFR, body mass index level, HDL cholesterol level, total cholesterol level, hemoglobin A1c, and smoking status | | | | | |  |  |
| ‡Unable to estimate variance due to inadequate sample size | | | | | |  |  |
