# Supplementary material for: Dose-Response Association of Uncontrolled Blood Pressure and Cardiovascular Disease Risk Factors with Hyperuricemia and Gout
Source: PLoS One. 2013 Feb 27;8(2):e56546. doi: 10.1371/journal.pone.0056546 (PMC3584090; doi:10.1371/journal.pone.0056546)
Supplement: Table S3 — Prevalence of Hyperuricemia by Level of Cardiovascular Disease Risk Factor in NHANES 2003–2006. (DOCX) [file pone.0056546.s003.docx]

| **Supplemental Table S3. Prevalence of Hyperuricemia by Level of Cardiovascular Disease Risk Factor in NHANES 2003-2006** | | | | | | |
| --- | --- | --- | --- | --- | --- | --- |
|  |  |  | NHANES 2003-2006 | |  | |
|  |  | Unweighted No. | Prevalence, % (SE) | Partially Adjusted Prevalence Ratio (95% CI)* | Fully Adjusted Prevalence Ratio (95% CI)† | |
| Blood Pressure (mmHg) | |  |  |  |  | |
|  | SBP<120 or DBP<80 | 4,416 | 11.25 (0.65) | Ref | Ref | |
|  | SBP: 120-139 or DBP: 80-89 | 3,216 | 19.11 (0.90) | 1.43 (1.22, 1.67) | 1.19 (1.04, 1.37) | |
|  | SBP: 140-159 or DBP: 90-99 | 1,219 | 23.70 (1.85) | 1.59 (1.27, 1.99) | 1.25 (1.01, 1.55) | |
|  | SBP ≥160 or DBP ≥100 | 589 | 25.91 (2.08) | 1.69 (1.36, 2.11) | 1.43 (1.13, 1.80) | |
| Body Mass Index (kg/m^2^) | |  |  |  |  | |
|  | <18.5 | 180 | 4.98 (1.90) | 0.74 (0.32, 1.72) | 0.82 (0.33, 2.05) | |
|  | 18.5-24.9 | 3,073 | 7.33 (0.61) | Ref | Ref | |
|  | 25-29.9 | 3,299 | 15.70 (0.96) | 1.89 (1.56, 2.28) | 1.61 (1.33, 1.96) | |
|  | 30-34.9 | 1,902 | 24.34 (1.02) | 3.02 (2.52, 3.61) | 2.43 (2.03, 2.91) | |
|  | ≥35 | 1,323 | 31.25 (1.68) | 4.17 (3.43, 5.07) | 3.34 (2.76, 4.03) | |
| Estimated GFR (mL/min per 1.73m^2^) | |  |  |  |  | |
|  | ≥90 | 5,874 | 11.39 (0.58) | Ref | Ref | |
|  | 60-89 | 3,090 | 19.10 (0.81) | 1.64 (1.45, 1.86) | 1.60 (1.43, 1.78) | |
|  | 30-59 | 881 | 42.75 (2.36) | 3.80 (3.22, 4.48) | 3.82 (3.22, 4.52) | |
|  | 15-29 | 79 | 74.58‡ | 6.54 (5.10, 8.38) | 6.97 (5.13, 9.48) | |
| HDL Cholesterol (mg/dL) | |  |  |  |  | |
|  | Men or Women ≥60 | 3,258 | 10.56 (0.68) | Ref | Ref | |
|  | Men 40-59; Women 50-59 | 4,124 | 17.11 (0.80) | 1.47 (1.24, 1.75) | 1.21 (1.01, 1.45) | |
|  | Men <40, Women <50 | 2,557 | 23.16 (1.22) | 2.20 (1.85, 2.63) | 1.54 (1.30, 1.82) | |
| Total Cholesterol (mg/dL) | |  |  |  |  | |
|  | <200 | 5,553 | 14.74 (0.73) | Ref | Ref | |
|  | 200-239 | 2,862 | 17.28 (1.10) | 1.11 (0.94, 1.32) | 1.17 (0.98, 1.40) | |
|  | ≥240 | 1,525 | 21.81 (1.66) | 1.40 (1.18, 1.67) | 1.37 (1.14, 1.64) | |
| Hemoglobin A1c (%), % | |  |  |  |  | |
|  | Normal (<5.7) | 7,959 | 14.63 (0.64) | Ref | Ref | |
|  | Prediabetes (5.7-6.4) | 1,169 | 30.43 (1.77) | 1.66 (1.43, 1.92) | 1.22 (1.05, 1.43) | |
|  | Diabetes (≥6.5) | 795 | 22.23 (2.05) | 1.25 (1.01, 1.55) | 0.84 (0.68, 1.03) | |
| Smoking Status, % | |  |  |  |  | |
|  | Never | 4,573 | 16.17 (0.68) | Ref | Ref | |
|  | Former | 2,352 | 20.85 (1.15) | 1.05 (0.93, 1.19) | 1.14 (1.00, 1.30) | |
|  | Current | 1,984 | 14.15 (0.98) | 0.88 (0.75, 1.04) | 1.04 (0.88, 1.23) | |
| Abbreviations: GFR, glomerular filtration rate; HDL, high density lipoprotein | | | | | | |
| *Adjusted for age, gender, and race/ethnicity | | | | | |  |
| †Adjusted for age, gender, race/ethnicity, blood pressure level, estimated GFR, body mass index level, HDL cholesterol level, total cholesterol level, hemoglobin A1c, and smoking status | | | | | |  |
| ‡Unable to estimate variance due to inadequate sample size | | | | | |  |
