# Supplementary material for: Dose-Response Association of Uncontrolled Blood Pressure and Cardiovascular Disease Risk Factors with Hyperuricemia and Gout
Source: PLoS One. 2013 Feb 27;8(2):e56546. doi: 10.1371/journal.pone.0056546 (PMC3584090; doi:10.1371/journal.pone.0056546)
Supplement: Table S4 — Prevalence of Hyperuricemia by Level of Cardiovascular Disease Risk Factor in NHANES 2007–2010. (DOCX) [file pone.0056546.s004.docx]

| **Supplemental Table S4. Prevalence of Hyperuricemia by Level of Cardiovascular Disease Risk Factor in NHANES 2007-2010** | | | | | |
| --- | --- | --- | --- | --- | --- |
|  |  |  | NHANES 2007-2010 | |  |
|  |  | Unweighted No. | Prevalence, % (SE) | Partially Adjusted Prevalence Ratio (95% CI)* | Fully Adjusted Prevalence Ratio (95% CI)† |
| Blood Pressure (mmHg) | |  |  |  |  |
|  | SBP<120 or DBP<80 | 5,123 | 13.47 (0.63) | Ref | Ref |
|  | SBP: 120-139 or DBP: 80-89 | 3,928 | 21.31 (0.94) | 1.35 (1.21, 1.52) | 1.17 (1.05, 1.31) |
|  | SBP: 140-159 or DBP: 90-99 | 1,470 | 25.98 (1.65) | 1.51 (1.24, 1.84) | 1.26 (1.04, 1.53) |
|  | SBP≥160 or DBP ≥100 | 542 | 30.16‡ | 1.67 (1.34, 2.07) | 1.46 (1.18, 1.80) |
| Body Mass Index (kg/m^2^) | |  |  |  |  |
|  | <18.5 | 199 | 1.68 (0.72) | 0.23 (0.09, 0.56) | 0.27 (0.11, 0.67) |
|  | 18.5-24.9 | 3,190 | 7.78 (0.59) | Ref | Ref |
|  | 25-29.9 | 3,855 | 16.14 (0.91) | 1.87 (1.55, 2.25) | 1.71 (1.39, 2.10) |
|  | 30-34.9 | 2,347 | 27.04 (1.52) | 3.13 (2.58, 3.78) | 2.72 (2.28, 3.24) |
|  | ≥35 | 1,783 | 35.13 (1.62) | 4.37 (3.67, 5.20) | 3.63 (3.07, 4.30) |
| Estimated GFR (mL/min per 1.73m^2^) | |  |  |  |  |
|  | ≥90 | 7,040 | 12.78 (0.62) | Ref | Ref |
|  | 60-89 | 3,485 | 23.10 (0.75) | 1.93 (1.63, 2.27) | 1.87 (1.62, 2.16) |
|  | 30-59 | 887 | 49.02‡ | 4.00 (3.27, 4.90) | 4.26 (3.53, 5.14) |
|  | 15-29 | 81 | 70.40‡ | 6.50 (4.76, 8.87) | 5.99 (4.38, 8.20) |
| HDL Cholesterol (mg/dL) | |  |  |  |  |
|  | Men or Women ≥60 | 3,232 | 11.60 (0.74) | Ref | Ref |
|  | Men 40-59; Women 50-59 | 4,459 | 17.54 (0.81) | 1.38 (1.18, 1.61) | 1.13 (0.96, 1.33) |
|  | Men <40, Women <50 | 3,834 | 25.13 (1.03) | 2.17 (1.90, 2.47) | 1.43 (1.25, 1.63) |
| Total Cholesterol (mg/dL) | |  |  |  |  |
|  | <200 | 6,679 | 16.58 (0.66) | Ref | Ref |
|  | 200-239 | 3,277 | 19.99 (0.92) | 1.18 (1.07, 1.31) | 1.25 (1.12, 1.39) |
|  | ≥240 | 1,569 | 22.09 (1.57) | 1.27 (1.07, 1.50) | 1.35 (1.16, 1.59) |
| Hemoglobin A1c (%) | |  |  |  |  |
|  | Normal (<5.7) | 7,889 | 15.49 (0.61) | Ref | Ref |
|  | Prediabetes (5.7-6.4) | 2,384 | 26.41 (1.22) | 1.45 (1.32, 1.60) | 1.11 (1.01, 1.21) |
|  | Diabetes (≥6.5) | 1,225 | 28.33 (1.59) | 1.49 (1.29, 1.73) | 0.90 (0.79, 1.02) |
| Smoking Status | |  |  |  |  |
|  | Never | 5,829 | 17.67 (0.59) | Ref | Ref |
|  | Former | 2,745 | 22.81 (1.33) | 1.08 (0.94, 1.24) | 1.11 (0.99, 1.25) |
|  | Current | 2,415 | 15.76 (0.88) | 0.90 (0.79, 1.01) | 1.02 (0.92, 1.13) |
| Abbreviations: GFR, glomerular filtration rate; HDL, high density lipoprotein; NA, not available | | | | | |
| *Adjusted for age, gender, and race/ethnicity | | | | | |
| †Adjusted for age, gender, race/ethnicity, blood pressure level, estimated GFR, body mass index level, HDL cholesterol level, total cholesterol level, hemoglobin A1c, and smoking status | | | | | |
| ‡Unable to estimate variance due to inadequate sample size | | | | | |
|  | | | | | |
