# Supplementary material for: Dose-Response Association of Uncontrolled Blood Pressure and Cardiovascular Disease Risk Factors with Hyperuricemia and Gout
Source: PLoS One. 2013 Feb 27;8(2):e56546. doi: 10.1371/journal.pone.0056546 (PMC3584090; doi:10.1371/journal.pone.0056546)
Supplement: Table S5 — Prevalence of Gout Defined by Self-Report and Either Hyperuricemia or Gout Medication Use According to Number of Cardiovascular Disease Risk Factors. (DOCX) [file pone.0056546.s005.docx]

| **Supplemental Table S5. Prevalence of Gout Defined by Self-Report and Either Hyperuricemia or Gout Medication Use According to Number of Cardiovascular Disease Risk Factors** | | | | |  |
| --- | --- | --- | --- | --- | --- |
|  |  | Unweighted No.* | Prevalence (SE) | Prevalence Ratio† | *P* |
| NHANES 1988-1994 | |  |  |  |  |
|  | Healthy‡ | 5,486 | 0.51 (0.21) | Ref | Ref |
|  | Uncontrolled BP Alone§ | 973 | 2.53 (0.88) | 2.06 (0.71, 6.00) | 0.18 |
|  | Plus 1 CVD Risk Factorǁ | 1,488 | 4.03 (0.90) | 3.47 (1.39, 8.67) | <0.01 |
|  | Plus 2 CVD Risk Factors | 986 | 5.86 (1.27) | 5.87 (2.47, 13.91) | <0.01 |
| NHANES 2007-2010 | |  |  |  |  |
|  | Healthy‡ | 3,324 | 0.69 (0.12) | Ref | Ref |
|  | Uncontrolled BP Alone§ | 564 | 1.63 (0.43) | 1.07 (0.57, 2.01) | 0.83 |
|  | Plus 1 CVD Risk Factorǁ | 783 | 4.69 (0.77) | 3.37 (1.85, 6.15) | <0.01 |
|  | Plus 2 CVD Risk Factors | 523 | 9.22¶ | 8.41 (4.86, 14.52) | <0.01 |
| Abbreviations: BP, blood pressure; CVD, cardiovascular disease | | |  |  |  |
| *The unweighted total number of people (denominator) available in each category | | | | | |
| †Adjusted for age, gender, and race/ethnicity | | | | | |
| ‡Healthy is defined as the absence of uncontrolled blood pressure and any of the 4 cardiovascular disease risk factors associated with serum uric acid | | | | | |
| §Uncontrolled blood pressure, defined as a systolic blood pressure ≥140 mmHg or diastolic blood pressure ≥90 mmHg, with no additional cardiovascular disease risk factors | | | | | |
| ǁA cardiovascular disease risk factor is defined as any of the following: estimated glomerular filtration rate <60 mL/min per 1.73m^2^, body mass index ≥30 kg/m^2^, high density lipoprotein <40 mg/dL in men or <50 mg/dL in women, or total cholesterol ≥240 mg/dL | | | | | |
| ¶Unable to estimate variance due to inadequate sample size | | | | | |
|  | | | | | |
