# Supplementary material for: Dose-Response Association of Uncontrolled Blood Pressure and Cardiovascular Disease Risk Factors with Hyperuricemia and Gout
Source: PLoS One. 2013 Feb 27;8(2):e56546. doi: 10.1371/journal.pone.0056546 (PMC3584090; doi:10.1371/journal.pone.0056546)
Supplement: Table S6 — Prevalence of Hyperuricemia According to Number of Cardiovascular Disease Risk Factors. (DOCX) [file pone.0056546.s006.docx]

| **Supplemental Table S6. Prevalence of Hyperuricemia According to Number of Cardiovascular Disease Risk Factors*** | | | | | |
| --- | --- | --- | --- | --- | --- |
|  |  | Unweighted No.† | Prevalence (SE) | Prevalence Ratio‡ | *P*-value |
| NHANES 1988-1994 | |  |  |  |  |
|  | Healthy§ | 5,499 | 6.60 (0.49) | Ref | Ref |
|  | 1 CVD Risk Factor | 5,399 | 13.67 (0.64) | 1.98 (1.69, 2.31) | <0.001 |
|  | 2 CVD Risk Factors | 3,632 | 25.36 (1.12) | 3.55 (3.02, 4.18) | <0.001 |
|  | 3 CVD Risk Factors | 1,300 | 38.57 (2.56) | 5.14 (4.29, 6.17) | <0.001 |
| NHANES 1999-2002 | |  |  |  |  |
|  | Healthy | 3,255 | 8.23 (0.56) | Ref | Ref |
|  | 1 CVD Risk Factor | 3,389 | 15.24 (0.98) | 1.81 (1.54, 2.13) | <0.001 |
|  | 2 CVD Risk Factors | 2,264 | 28.16 (1.54) | 3.35 (2.76, 4.05) | <0.001 |
|  | 3 CVD Risk Factors | 777 | 37.28 (2.78) | 4.30 (3.48, 5.32) | <0.001 |
| NHANES 2003-2006 | |  |  |  |  |
|  | Healthy | 3,590 | 7.21 (0.61) | Ref | Ref |
|  | 1 CVD Risk Factor | 3,475 | 15.99 (0.74) | 2.12 (1.71, 2.61) | <0.001 |
|  | 2 CVD Risk Factors | 2,145 | 27.20 (1.19) | 3.53 (2.93, 4.24) | <0.001 |
|  | 3 CVD Risk Factors | 613 | 36.15 (2.46) | 4.49 (3.48, 5.79) | <0.001 |
| NHANES 2007-2010 | |  |  |  |  |
|  | Healthy | 3,820 | 7.58 (0.63) | Ref | Ref |
|  | 1 CVD Risk Factor | 3,990 | 17.51 (0.81) | 2.20 (1.85, 2.60) | <0.001 |
|  | 2 CVD Risk Factors | 2,741 | 30.29 (1.26) | 3.79 (3.13, 4.59) | <0.001 |
|  | 3 CVD Risk Factors | 833 | 38.58ǁ^e^ | 4.61 (3.74, 5.69) | <0.001 |
| Abbreviations: CVD, cardiovascular disease | | | | | |
| *A cardiovascular disease risk factor is defined as any of the following: systolic blood pressure ≥140 mmHg or diastolic blood pressure ≥90 mmHg, estimated glomerular filtration rate <60 mL/min per 1.73m^2^, body mass index ≥30 kg/m^2^, high density lipoprotein <40 mg/dL in men or <50 mg/dL in women, or total cholesterol ≥240 mg/dL | | | | | |
| †The unweighted total number of people (denominator) available in each category | | | | | |
| ‡Adjusted for age, gender, and race/ethnicity | | | | | |
| §Healthy is defined as the absence of uncontrolled blood pressure and any of the 4 cardiovascular risk factors associated with serum uric acid | | | | | |
| ǁUnable to estimate variance due to inadequate sample size in several survey design strata | | | | |  |
|  | | | | |  |
