# Supplementary material for: Dose-Response Association of Uncontrolled Blood Pressure and Cardiovascular Disease Risk Factors with Hyperuricemia and Gout
Source: PLoS One. 2013 Feb 27;8(2):e56546. doi: 10.1371/journal.pone.0056546 (PMC3584090; doi:10.1371/journal.pone.0056546)
Supplement: Table S7 — Prevalence of Gout According to Number of Cardiovascular Disease Risk Factors. (DOCX) [file pone.0056546.s007.docx]

| **Supplemental Table S7. Prevalence of Gout According to Number of Cardiovascular Disease Risk Factors*** | | | | | |
| --- | --- | --- | --- | --- | --- |
|  |  | Unweighted No.† | Prevalence (SE) | Prevalence Ratio‡ | *P*-value |
| NHANES 1988-1994 | |  |  |  |  |
|  | Healthy§ | 5,498 | 1.03 (0.25) | Ref | Ref |
|  | 1 CVD Risk Factor | 5,399 | 1.68 (0.19) | 1.20 (0.75, 1.92) | 0.45 |
|  | 2 CVD Risk Factors | 3,632 | 4.93 (0.57) | 2.85 (1.68, 4.83) | <0.001 |
|  | 3 CVD Risk Factors | 1,300 | 7.73 (1.01) | 3.77 (2.18, 6.50) | <0.001 |
| NHANES 2007-2008 | |  |  |  |  |
|  | Healthy | 1,689 | 1.29 (0.20) | Ref | Ref |
|  | 1 CVD Risk Factor | 1,821 | 3.22 (0.44) | 2.02 (1.18, 3.49) | 0.014 |
|  | 2 CVD Risk Factors | 1,303 | 6.18 (0.81) | 3.80 (2.10, 6.88) | <0.001 |
|  | 3 CVD Risk Factors | 417 | 10.76 (1.50) | 5.74 (3.28, 10.05) | <0.001 |
| NHANES 2009-2010 | |  |  |  |  |
|  | Healthy | 1,794 | 1.65 (0.22) | Ref | Ref |
|  | 1 CVD Risk Factor | 2,036 | 3.17 (0.50) | 1.41 (0.89, 2.26) | 0.13 |
|  | 2 CVD Risk Factors | 1,373 | 5.72 (1.11) | 2.43 (1.35, 4.37) | 0.005 |
|  | 3 CVD Risk Factors | 408 | 9.62ǁ | 3.33 (2.11, 5.27) | <0.001 |
| NHANES 2007-2010 | |  |  |  |  |
|  | Healthy | 3,483 | 1.47 (0.15) | Ref | Ref |
|  | 1 CVD Risk Factor | 3,857 | 3.19 (0.33) | 1.68 (1.20, 2.35) | 0.004 |
|  | 2 CVD Risk Factors | 2,676 | 5.95 (0.68) | 3.00 (2.02, 4.44) | <0.001 |
|  | 3 CVD Risk Factors | 825 | 10.2ǁ | 4.36 (3.07, 6.19) | <0.001 |
| Abbreviations: CVD, cardiovascular disease | | | | | |
| *A cardiovascular disease risk factor is defined as any of the following: systolic blood pressure ≥140 mmHg or diastolic blood pressure ≥90 mmHg, estimated glomerular filtration rate <60 mL/min per 1.73m^2^, body mass index ≥30 kg/m^2^, high density lipoprotein <40 mg/dL in men or <50 mg/dL in women, or total cholesterol ≥240 mg/dL | | | | | |
| †The unweighted total number of people (denominator) available in each category | | | | |  |
| ‡Adjusted for age, gender, and race/ethnicity | | |  |  |  |
| §Healthy is defined as the absence of uncontrolled blood pressure and any of the 4 cardiovascular risk factors associated with serum uric acid | | | | | |
| ǁUnable to estimate variance due to inadequate sample size in several survey design strata | | | | | |
