# Supplementary material for: Dose-Response Association of Uncontrolled Blood Pressure and Cardiovascular Disease Risk Factors with Hyperuricemia and Gout
Source: PLoS One. 2013 Feb 27;8(2):e56546. doi: 10.1371/journal.pone.0056546 (PMC3584090; doi:10.1371/journal.pone.0056546)
Supplement: Table S8 — Prevalence Ratios of Gout by Strata of Gender or Race/Ethnicity in NHANES 1988–1994 & 2007–2010. (DOCX) [file pone.0056546.s008.docx]

| **Supplemental Table S8. Prevalence Ratios of Gout by Strata of Gender or Race/Ethnicity in NHANES 1988-1994 & 2007-2010** | | | | |
| --- | --- | --- | --- | --- |
|  |  | Prevalence Ratio (95% CI)* | | |
| Female | | NHANES 1988-1994 |  | NHANES 2007-2010 |
|  | Healthy‡ | Ref |  | Ref |
|  | Uncontrolled BP Alone§ | 3.03 (0.71, 13.03) |  | 0.37 (0.07, 1.93) |
|  | Plus 1 CVD Risk Factorǁ | 4.22 (1.35, 13.12) |  | 2.42 (1.01, 5.79) |
|  | Plus 2 CVD Risk Factors | 5.51 (1.45, 20.91) |  | 4.37 (1.53, 12.54) |
| Male | |  |  |  |
|  | Healthy‡ | Ref |  | Ref |
|  | Uncontrolled BP Alone§ | 1.65 (0.83, 3.28) |  | 1.76 (0.91, 3.40) |
|  | Plus 1 CVD Risk Factorǁ | 2.30 (1.26, 4.22) |  | 2.43 (1.51, 3.90) |
|  | Plus 2 CVD Risk Factors | 3.56 (1.94, 6.52) |  | 4.54 (2.99, 6.90) |
| Non-Hispanic white | |  |  |  |
|  | Healthy‡ | Ref |  | Ref |
|  | Uncontrolled BP Alone§ | 1.97 (1.08, 3.58) |  | 1.44 (0.68, 3.02) |
|  | Plus 1 CVD Risk Factorǁ | 2.77 (1.54, 4.97) |  | 2.04 (1.08, 3.86) |
|  | Plus 2 CVD Risk Factors | 3.75 (1.99, 7.06) |  | 3.91 (2.79, 5.49) |
| Non-Hispanic black | |  |  |  |
|  | Healthy‡ | Ref |  | Ref |
|  | Uncontrolled BP Alone§ | 1.36 (0.50, 3.68) |  | 1.54 (0.45, 5.22) |
|  | Plus 1 CVD Risk Factorǁ | 1.90 (0.78, 4.62) |  | 4.46 (2.05, 9.68) |
|  | Plus 2 CVD Risk Factors | 2.69 (1.01, 7.12) |  | 4.74 (1.83, 12.23) |
| Mexican American | |  |  |  |
|  | Healthy‡ | Ref |  | Ref |
|  | Uncontrolled BP Alone§ | 4.52 (0.69, 29.63) |  | 0.00† |
|  | Plus 1 CVD Risk Factorǁ | 1.68 (0.33, 8.52) |  | 3.23 (0.79, 13.23) |
|  | Plus 2 CVD Risk Factors | 10.54 (1.82, 61.02) |  | 9.09 (1.72, 47.92) |
| *Adjusted for age and either gender or race/ethnicity depending on the strata | | | | |
| ‡Healthy is defined as the absence of uncontrolled blood pressure and any of the 4 cardiovascular disease risk factors associated with serum uric acid | | | | |
| §Uncontrolled blood pressure, defined as a systolic blood pressure ≥140 mmHg or diastolic blood pressure ≥90 mmHg, with no additional cardiovascular disease risk factors | | | | |
| ǁA cardiovascular disease risk factor is defined as any of the following: estimated glomerular filtration rate <60 mL/min per 1.73m^2^, body mass index ≥30 kg/m^2^, high density lipoprotein <40 mg/dL (1.04 mmol/L) in men or <50 mg/dL (1.30 mmol/L) in women, or total cholesterol ≥240 mg/dL (6.22 mmol/L)  †There were no cases of gout in this stratum. | | | | |
